# Tracking Technological Change in Liver Surgery: Robotic Adoption and Case-Mix Evolution in the I Go MILS Registry

**DOI:** 10.3390/cancers18152516

**Published:** 2026-08-06

**Authors:** Luca Ruotolo, Alessandro Ferrero, Andrea Ruzzenente, Felice Giuliante, Vincenzo Mazzaferro, Umberto Cillo, Salvatore Gruttadauria, Fabrizio Di Benedetto, Giorgio Ercolani, Matteo Ravaioli, Giuseppe Maria Ettorre, Andrea Belli, Giovanni Vennarecci, Raffaele Dalla Valle, Elio Jovine, Francesca Ratti, on behalf of the I Go MILS Collaborative Group

**Affiliations:** 1 Hepatobiliary Surgery Division, IRCCS San Raffaele Scientific Institute, Via Olgettina 60, 20132 Milano, Italy; ruotolo.luca@hsr.it; 2 Department of Surgery, Mauriziano Hospital, 10128 Turin, Italy; aferrero@mauriziano.it; 3 Division of General and Hepatobiliary Surgery, Department of Surgery, Dentistry, Gynecology, and Pediatrics, G.B. Rossi University Hospital, University of Verona l, 37134 Verona, Italy; andrea.ruzzenente@univr.it; 4 Hepatobiliary Surgery Unit, Fondazione Policlinico Universitario A. Gemelli IRCSS, Università Cattolica del Sacro Cuore, 00168 Rome, Italy; felice.giuliante@unicatt.it; 5 Department of Surgery, Division of HPB Surgery and Liver Transplantation, Fondazione IRCCS Istituto Nazionale Tumori Di Milano, 20133 Milan, Italy; vincenzo.mazzaferro@istitutotumori.mi.it; 6 Department of Surgery, Oncology and Gastroenterology (DiSCOG), University of Padua, 35128 Padua, Italy; cillo@unipd.it; 7 Hepato-Bilio-Pancreatic Surgery and Liver Transplantation Unit, Azienda Ospedale Universita Padova, 35128 Padua, Italy; 8 Clinical Pharmacy Service, IRCCS ISMETT (Istituto Mediterraneo per i Trapianti e Terapie ad Alta Specializzazione), UPMC (University of Pittsburgh Medical Center), 90127 Palermo, Italy; sgruttadauria@ismett.edu; 9 Abdominal Surgery and Organ Transplantation Unit, Department for the Treatment and Study of Abdominal Diseases and Abdominal Transplantation, IRCCS ISMETT (Istituto Mediterraneo per i Trapianti e Terapie ad Alta Specializzazione), UPMC (University of Pittsburgh Medical Center), 90127 Palermo, Italy; 10 Hepato-Pancreato-Biliary Surgery and Liver Transplantation Unit, University Hospital of Modena “Policlinico”, University of Modena and Reggio Emilia, 41124 Modena, Italy; fabrizio.dibenedetto@unimore.it; 11 Department of Medical and Surgical Sciences, Alma Mater Studiorum, University of Bologna, 40138 Bologna, Italy; giorgio.ercolani2@unibo.it (G.E.); matteo.ravaioli6@unibo.it (M.R.); elio.jovine@aosp.bo.it (E.J.); 12 General and Oncology Surgery, Morgagni-Pierantoni Hospital, Ausl Romagna, Forlì 47121, Italy; 13 Hepato-Biliary and Transplant Unit, IRCCS Azienda Ospedaliero–Universitaria di Bologna, 40138 Bologna, Italy; 14 Department of Surgery, San Camillo Forlanini Hospital, 00152 Rome, Italy; gmettorre@scamilloforlanini.rm.it; 15 Department of Gastro-Hepato-Pancreato-Biliary Surgery, Istituto Nazionale Tumori, IRCCS-Fondazione “G. Pascale”, Via Mariano Semmola, 80131 Napoli, Italy; a.belli@istitutotumori.na.it; 16 Department of Hepatobiliary and Liver Transplantation Surgery, Azienda Ospedaliera di Rilievo Nazionale (A.O.R.N). Cardarelli, 80131 Naples, Italy; giovanni.vennarecci@aocardarelli.it; 17 Hepatobiliary Surgery Unit, Department of Medicine and Surgery, University of Parma, 43126 Parma, Italy; raffaele.dallavalle@unipr.it; 18 Department of General Surgery, IRCCS Azienda Ospedaliero-Universitaria Di Bologna, 40138 Bologna, Italy; 19 Faculty of Medicine and Surgery, Vita-Salute San Raffaele University, 20134 Milan, Italy

**Keywords:** minimally invasive, laparoscopic, robotic, registry

## Abstract

**Background**: The adoption of robotic surgery in minimally invasive liver surgery has accelerated globally in the last decade, yet its impact on clinical outcomes and the associated benefits remain to be explored. **Methods**: The study analyzed 8928 minimally invasive liver resections recorded in the I Go MILS registry between 2015 and 2025. Temporal trends in surgical approach, Pringle maneuver utilization, conversion to open surgery, and postoperative morbidity were assessed using Cochran–Armitage and Spearman tests. Case-mix evolution was quantified by the Kawaguchi–Gayet difficulty score. **Results**: Robotic utilization increased monotonically from 10.4% to 42.2%, while fully laparoscopic procedures declined from 89.6% to 57.8%. Despite this transition, aggregate conversion and morbidity showed no significant temporal trends. Stratified analysis demonstrated lower observed conversion rates in the robotic group and a progressively narrowing difference in morbidity. Overall case-mix complexity increased significantly, driven exclusively by the laparoscopic arm, while the robotic case mix remained structurally stable. **Conclusions**: Aggregate outcome stability during the robotic transition suggests progressive case-mix complexification rather than technological stagnation. The robotic platform seems to be associated with an apparent conversion advantage; its higher observed morbidity may be attributable to structural differences in operative difficulty. Robotics act not as a substitute for laparoscopy but as a complementary approach applied to more complex resections.

## Simple Summary

This study looked at nearly 9000 minimally invasive liver surgeries recorded over a ten-year period to understand how the growing use of robotic surgery has been affecting patient outcomes compared to traditional laparoscopic surgery. The aim was to find out whether the rise in robotic procedures was truly improving results, or whether other factors were influencing the data. Although robotic surgery became much more common, overall rates of complications and the need to switch to open surgery stayed stable. This was because laparoscopic surgery increasingly took on more complex cases, while robotic surgery consistently showed an apparent advantage in avoiding conversion. These findings suggest that robotic surgery is not simply replacing laparoscopic techniques, but is being used as a complementary tool for more challenging operations. This insight can help surgeons and researchers better understand how to select the right approach for each patient and guide future improvements in surgical training.

## 1. Introduction

Minimally invasive liver surgery (MILS) has progressively evolved from a selected approach for minor peripheral resections to an established component of modern hepatobiliary surgery. This expansion has been supported by international consensus statements and guidelines, which have emphasized structured training, appropriate patient selection, technical standardization, and continuous outcome monitoring during the adoption of more complex procedures [1,2,3,4]. In parallel, the increasing diffusion of robotic platforms has introduced a further technological shift within MILS, offering potential advantages in visualization, instrument articulation, ergonomics, and precision during complex liver resections.

In rapidly evolving surgical fields, registries are essential not only to report outcomes, but also to capture real-world implementation and track temporal trends in clinical practice. The Italian Group of Minimally Invasive Liver Surgery—I Go MILS—was established as a national registry to monitor the diffusion, indications, outcomes, and evolution of MILS in Italy. Since its first report, I Go MILS has generated several analyses on national implementation, center-volume effects, risk-adjusted outcomes, benchmarks, and indication-specific results, confirming its role as a dynamic platform for dissemination, characterization, and networking [5,6].

Robotic liver surgery represents one of the most relevant changes in the recent evolution of MILS [7,8,9,10,11]. Previous studies have suggested that robotic strategy may optimize intraoperative performance in technically demanding liver resections, including major hepatectomies, complex parenchymal transections, and procedures requiring lymphadenectomy [12,13,14,15]. However, the interpretation of robotic adoption requires longitudinal evaluation. A simple comparison between robotic and laparoscopic surgery may be misleading if changes in indication, case mix, and procedural complexity over time are not considered.

The I Go MILS registry offers a unique opportunity to explore this issue over twelve years of national experience. By capturing the progressive redistribution between fully laparoscopic and robotic approaches, together with operative outcomes and procedural complexity, the registry allows the evaluation of how MILS practice has changed over time and whether robotics is emerging as a substitute for laparoscopy or rather as a complementary platform for more complex indications.

The primary aim of the present study was to describe and quantify the temporal evolution of robotic and fully laparoscopic liver surgery within the I Go MILS national registry over an eleven-year period.

The secondary aims were to evaluate whether increasing robotic adoption was associated with changes in key outcomes, including Pringle maneuver use, conversion to open surgery, and postoperative morbidity; to compare temporal outcome trajectories between robotic and fully laparoscopic resections; and to assess whether changes in procedural complexity may explain the relationship between technological adoption and aggregate outcomes.

## 2. Materials and Methods

### 2.1. Registry Presentation and Selection Criteria

The I Go MILS registry is built within a multicenter, prospective, observational, spontaneous study involving patients undergoing minimally invasive liver resection. Since 2014, 9791 patients were enrolled by 66 participating centers across Italy including both university and hospital-based surgical units.

For this specific analysis, patients were selected by liver resection approach (laparoscopic and/or robotic), with no specific discrimination of liver resection and clinical conditions, as part of an approach entirely focused on the intention-to-treat principle. The inclusion criteria required participants to be at least 18 years of age and to have signed the relevant informed consent form of the study. Patients who underwent procedures performed exclusively for diagnostic purposes and non-resective therapeutic procedures with minimally invasive approach were excluded from the study.

Instead, the study selected to analyze the data collected for full years, namely from 2015 to 2025, as the data for 2014 and 2026 are incomplete and do not represent full years. The selected period yielded a total analytical cohort of 8928 procedures.

The primary analysis involves assessing trends in robotic and laparoscopic procedures over an eleven-year period. Conversion rate to open surgery, utilization rate of Pringle maneuver and postoperative morbidity rate were considered to determine the effect of approach evolution. Each factor was assessed both by examining the overall trend and by analyzing the data stratified by approach.

Furthermore, the analysis also included trends in annual surgical difficulty to assess whether there were any correlations or potential cause–effect relationships in the preferential adoption of one of the two approaches. Operative difficulty was classified according to the Kawaguchi–Gayet scoring system, a validated instrument for the preoperative stratification of minimally invasive liver resection difficulty based on anatomical and technical criteria including lesion location by liver segment, lesion size, proximity to major hepatic vasculature, and resection type. The score assigns each procedure to one of three difficulty levels: Grade I (low difficulty), Grade II (intermediate difficulty), and Grade III (high difficulty) [16]. This system was originally developed and validated in the context of laparoscopic liver surgery and is applied here to both robotic and laparoscopic procedures, with the acknowledged limitation that the instrument does not formally account for technique-specific ergonomic advantages of the robotic platform.

Missing data relating to one or more of the variables (or outside the chosen timeframe) led to the exclusion of the entire records from the main analysis.

### 2.2. Statistics

Temporal trends in robotic and laparoscopic utilization, Pringle maneuver rate, conversion to open surgery, postoperative morbidity and case-mix evolution over time were assessed using a dual-test strategy combining the Cochran–Armitage test and the Spearman rank correlation, applied to annual clusters, while Fisher’s Chi-squared test with Monte Carlo simulation (B = 5000 permutations) was employed for the global distributional comparison of Kawaguchi–Gayet difficulty categories across temporal clusters and across surgical approaches.

All statistical analyses were performed in R (version ≥ 4.3). Temporal trend tests (Cochran–Armitage, Spearman) were implemented using the coin, stats, and DescTools packages. LOESS smoothing and volume-weighted smooth curves were generated using ggplot2 with the geom_smooth function. Fisher’s exact test with Monte Carlo simulation was performed using the base R fisher.test function with the simulate. *p*.value = TRUE argument and B = 5000 replicates. All figures were produced using ggplot2 (version ≥ 3.4). A significance threshold of α = 0.05 (two-sided) was applied throughout. No corrections for multiple comparisons were applied, given the exploratory and descriptive nature of the analysis; all *p*-values are therefore reported as unadjusted and should be interpreted accordingly.

## 3. Results

### 3.1. Registry Overview and Annual Surgical Volume

Between 2015 and 2025, a total of 8928 minimally invasive liver resections were recorded in the I Go MILS registry across participating centers. These procedures involved a large cohort of patients suffering from hepatocellular carcinoma (36.2%), liver metastasis (35.3%), benign conditions (18.6%), intrahepatic cholangiocarcinoma (6.5%) and other primary tumors (3.4%).

Annual procedural volume increased substantially during the first half of the observation period, rising from almost 500 resections in 2015 to a first peak of 1048 liver surgeries in 2019, followed by a 4-year peak operating period with around a thousand patients recruited yearly. A marked decrease in recorded volume was observed in 2024 (*n* = 836) and 2025 (*n* = 458), which stands in contrast to the genuine registry expansion and increased center participation reported in previous year (Table 1).

Although the study did not include a dedicated in-depth temporal analysis, key information about perioperative outcomes, mean operative time, blood loss, length of hospital stays, previous surgeries, mortality and complications severity are set out to provide a comprehensive context in which the study was carried out (Table 2).

**Figure 1:**
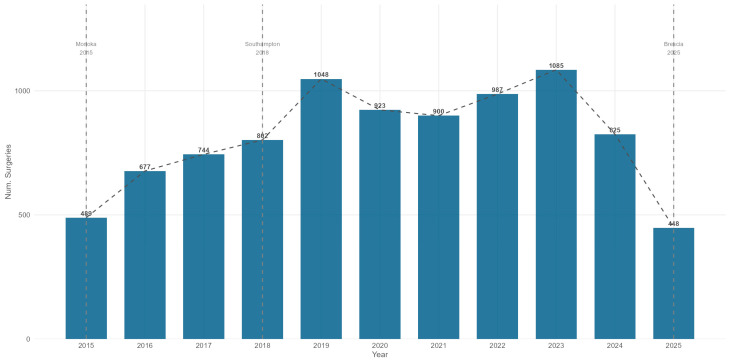
Annual volume of minimally invasive liver resections recorded in the IGoMILS registry (2015–2025; *N* = 8928). Each bar represents total procedures per calendar year. The dashed line connects annual values to highlight volumetric dynamics. Vertical dashed lines indicate three major international consensus milestones: Morioka (2015), Southampton (2018), and Brescia (2025).

Three major international consensus milestones in minimally invasive liver surgery are annotated as temporal reference points throughout the analysis: the Morioka Conference (2015), the Southampton Guidelines (2018), and the Brescia IEGUMILS meeting (2025) (Figure 1).

### 3.2. Temporal Trends in Liver Resection Technology Adoption

To contextualize these volumetric dynamics, a stratified analysis was conducted to identify the actual distribution of surgical approaches and their evolution over the study period.

**Figure 2:**
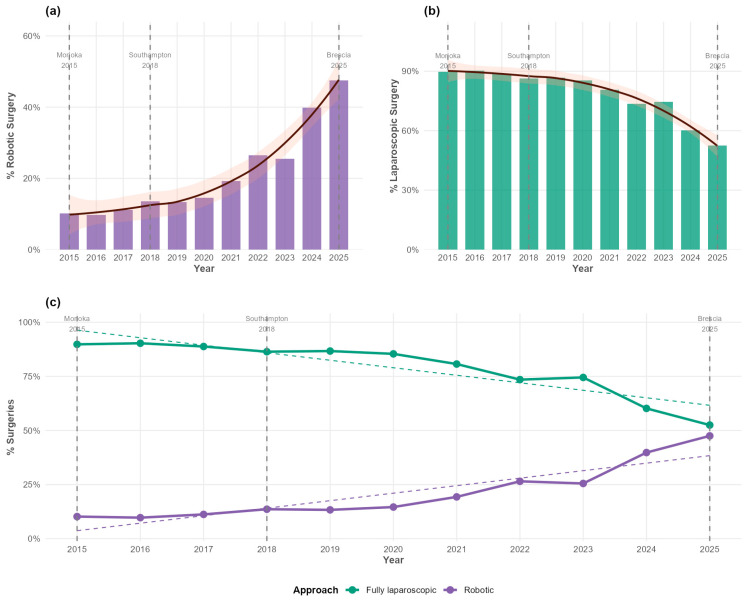
Temporal evolution of surgical approach distribution (2015–2025). (**a**) Annual proportion of robotic procedures, with LOESS-smoothed curve and 95% CI (black trend lines and orange shading) (Cochran–Armitage *p* < 0.001; Spearman ρ = 0.973, *p* < 0.001). (**b**) Annual proportion of fully laparoscopic procedures (Cochran–Armitage *p* < 0.001; Spearman ρ = −0.973, *p* < 0.001). (**c**) Year-by-year comparison of robotic vs. fully laparoscopic proportions with linear regression fits (dashed lines); both Cochran–Armitage *p* < 0.001. Vertical dashed lines: Morioka (2015), Southampton (2018), Brescia (2025).

The proportion of robotic procedures (Figure 2a) increased monotonically across the entire period starting from 10% in frequency in 2015 and reaching over 40% of LLRs eleven years later. The Cochran–Armitage test confirmed a highly significant increasing linear trend (z = 22.87, *p* < 0.001), while Spearman correlation between robotic proportion and calendar year on the annual series was ρ = 0.973 (*p* < 0.001), indicating near-perfect monotonic growth over eleven years.

Conversely, the proportion of fully laparoscopic procedures (Figure 2b) declined significantly and monotonically across each year, from 89.6% in 2015 and falling below 60% of cases by 2025 (Cochran–Armitage z = −22.87, *p* < 0.001). The Spearman correlation for the laparoscopic series at the cluster level was ρ = −0.973 (*p* < 0.001) on the annual series, mirroring the robotic trajectory with opposite sign.

The simultaneous representation of both trends on the annual chart (Figure 2c) revealed an incipient crossover between the two series in 2025, where fully laparoscopic and robotic proportions approached approximate parity (~52% and ~48%, respectively).

### 3.3. Temporal Trends in Vascular Control Practice and Clinical Outcomes Stratified by Approach

Alongside this apparent technological convergence, the study focused on the temporal evolution of intraoperative vascular control strategies, as examined through both general and stratified trends of Pringle maneuver utilization by approach. Moreover, the analysis assessed whether the shift toward robotic surgery was accompanied by measurable changes in the primary clinical outcome metrics, such as conversion rate to open surgery and postoperative morbidity. As for the Pringle maneuver, the outcomes are presented in general terms and broken down by robotic vs. laparoscopic approach.

#### 3.3.1. Pringle Maneuver

**Figure 3:**
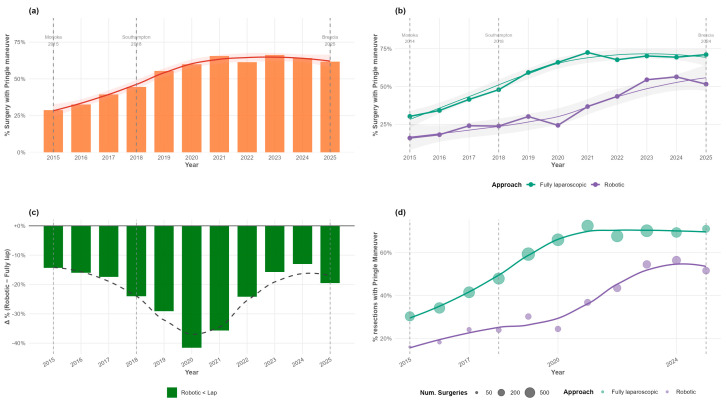
Temporal trends in Pringle maneuver utilization (2015–2025). (**a**) Aggregate annual proportion of procedures performed with Pringle maneuver; LOESS curve with 95% CI (red trend lines and orange shading) (Spearman ρ = 0.67, *p* = 0.012; Cochran–Armitage *p* < 0.001). (**b**) Annual Pringle rate stratified by approach (fully laparoscopic: ρ = 0.900, *p* < 0.001; robotic: ρ = 0.955, *p* < 0.001), with LOESS smooth curves and95% CI bands (green and violet shading and lines). (**c**) Year-by-year absolute difference (robotic – fully laparoscopic); all values negative throughout the observation period (black dashed line for trend). (**d**) Volume-weighted bubble chart with LOESS smooth curves by approach; bubble size proportional to annual caseload. Vertical dashed lines: Morioka (2015), Southampton (2018), Brescia (2025).

The utilization of the Pringle maneuver increased significantly across the study period, rising from 34.3% to a peak settling over 60% in 2021. On the annual series, the Spearman correlation was ρ = 0.67 (*p* = 0.012), a moderate value indicating a significant but non-perfectly monotonic relationship. Inspection of the LOESS-smoothed annual curve revealed a biphasic pattern: a rapid increase from approximately 31% in 2015 to a sustained plateau between 63% and 67% in 2020–2023. This discrepancy between the highly significant Cochran–Armitage result, which captures the dominant linear component across ordered clusters, and the moderate Spearman correlation, which is attenuated by the plateau, characterizes the Pringle trend as a sigmoid followed by potential stabilization rather than a purely linear increase (Figure 3a).

When stratified by surgical approach, both techniques showed statistically significant and highly concordant increasing trends in Pringle utilization, in contrast to the asymmetry observed in the preceding analytical version. For fully laparoscopic surgery, the Spearman correlation was ρ = 0.900 (*p* < 0.001) with Cochran–Armitage *p* < 0.001. For robotic surgery, the Spearman correlation was ρ = 0.955 (*p* < 0.001) with Cochran–Armitage *p* < 0.001. Both techniques therefore demonstrated near-perfect monotonic growth in Pringle utilization over the eleven-year period. Visual inspection of the stratified annual chart confirmed that the laparoscopic series started at approximately 30% in 2015 and reached approximately 70–73% by 2021–2023, then stabilized toward 70% in 2025, while the robotic series started at approximately 17% in 2015 and rose progressively to approximately 52–55% in 2024–2025 (Figure 3b). This evidence was supplemented by comparing the intervention volumes for each approach in each year to establish if the trend may be affected by the number of resections (Figure 3d). During the period under review, the number of fully laparoscopic resections was higher in absolute terms (around 500 per year) whilst the proportion of procedures performed robotically was generally lower. Nevertheless, the trends show a similar pattern of development, with laparoscopic procedures tending to be performed more frequently using the Pringle maneuver in each of the years considered.

The persistent inter-technique gap—with laparoscopic Pringle rates consistently exceeding robotic rates throughout the observation period—was confirmed by the delta analysis, which showed negative values (robotic < laparoscopic) in every year from 2015 to 2025. The maximum absolute gap was approximately −38 to −40 percentage points around 2019–2021, with a progressive but incomplete reduction in subsequent years as the robotic series continued to grow (Figure 3c).

#### 3.3.2. Conversion to Open Surgery

**Figure 4:**
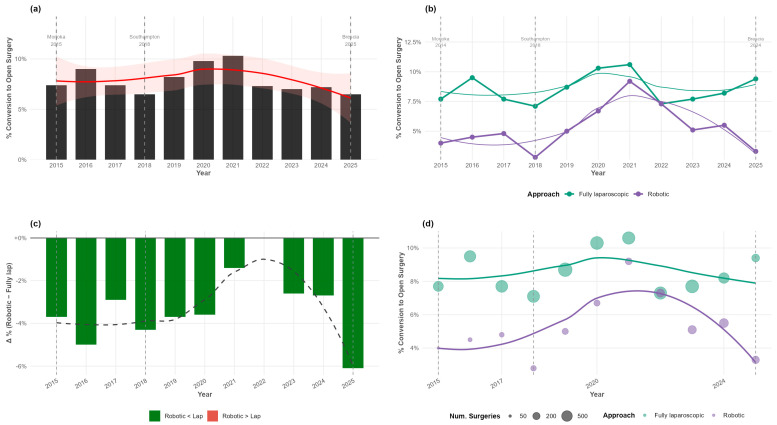
Temporal trends in conversion to open surgery (2015–2025). (**a**) Aggregate annual conversion rate; LOESS curve with 95% CI (red trend lines and orange shading) (Cochran–Armitage *p* = 0.391; Spearman ρ = −0.416, *p* = 0.204). (**b**) Annual conversion rate stratified by approach (robotic: ρ = 0.364, *p* = 0.272; fully laparoscopic: ρ = 0.128, *p* = 0.707), with 95% CI bands. (**c**) Year-by-year absolute difference (robotic – fully laparoscopic); green bars indicate robotic < laparoscopic in all years except one (black dashed trend line). (**d**) Volume-weighted bubble chart with LOESS smooth curves by approach. Vertical dashed lines: Morioka (2015), Southampton (2018), Brescia (2025).

The aggregate conversion rate showed no significant temporal trend across the study period (Figure 4a). On the annual series, Cochran–Armitage test yielded *p* = 0.391 and the Spearman correlation was ρ = −0.416 (*p* = 0.204) consistent with the absence of significant monotonic trend. The LOESS-smoothed annual curve revealed a non-linear pattern: values of approximately 7.5–8.5% in 2015–2019, a transient increase to approximately 10–11% in 2020–2021, and a return to approximately 7–8% in 2022–2025. The transient increase in 2020–2021 coincided with the COVID-19 pandemic period.

When analyzed separately by surgical approach (Figure 4b), neither robotic nor fully laparoscopic surgery demonstrated a statistically significant temporal trend in conversion rate. For robotic surgery on annual data, the Spearman correlation was ρ = 0.364 (*p* = 0.272) with Cochran–Armitage *p* = 0.964. For fully laparoscopic surgery, Spearman ρ = 0.128 (*p* = 0.707) with Cochran–Armitage *p* = 0.882. All tests were substantially non-significant for both techniques, confirming the absence of directional trend within each approach.

Notwithstanding this shared absence of temporal trend, a persistent and clinically meaningful inter-technique difference in conversion rates was documented across most of the time points through the analysis of delta percentages. This confirmed that robotic conversion was lower than laparoscopic in every year from 2015 to 2025, with the inter-technique gap ranging from approximately −1 to −6 percentage points annually, and the smoothed delta trend suggesting a widening of the advantage in the most recent years (Figure 4c). Furthermore, the number of resections performed each year does not appear to have influenced the conversion rate trends, which remain stable for each approach, although robotic resections are still less likely to result in conversion (Figure 4d).

#### 3.3.3. Postoperative Morbidity

**Figure 5:**
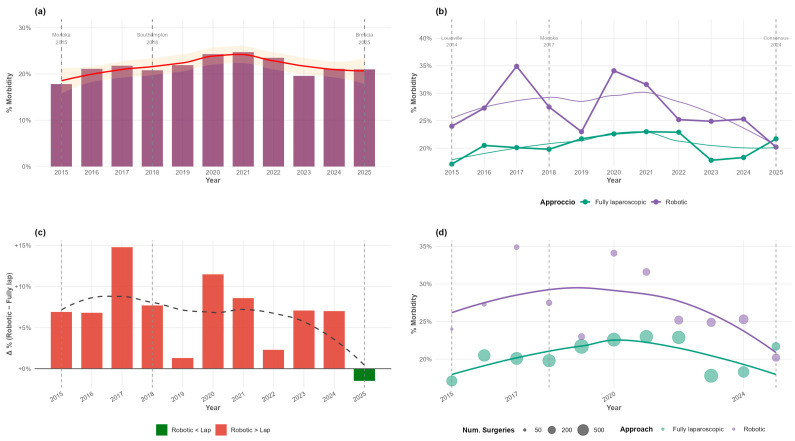
Temporal trends in postoperative morbidity (2015–2025). (**a**) Aggregate annual morbidity rate; LOESS curve with 95% CI (red trend lines and orange shading) (Cochran–Armitage *p* = 0.478; Spearman ρ = 0.137, *p* = 0.689). (**b**) Annual morbidity stratified by approach (robotic: Cochran–Armitage *p* = 0.046, Spearman ρ = −0.309, *p* = 0.355; fully laparoscopic: CA *p* = 0.649, ρ = 0.246, *p* = 0.466), with LOESS curves and 95% CI. (**c**) Year-by-year absolute difference (robotic – fully laparoscopic); red bars indicate robotic > laparoscopic throughout the observation period, with progressive convergence toward zero in 2025 (black dashed trend line). (**d**) Volume-weighted bubble chart with LOESS smooth curves by approach; bubble size proportional to annual caseload. Vertical dashed lines: Morioka (2015), Southampton (2018), Brescia (2025).

Postoperative morbidity showed no significant temporal trend at the annual level. The Cochran–Armitage test yielded *p* = 0.477 (z = 0.71), and the Spearman correlation was ρ = 0.137 (*p* = 0.689). The annual curve described an almost flat arch-shaped trajectory, with annual figures hovering around a stable figure of 20%, exceeded only during the pandemic years (2020–2022) (Figure 5a).

Conversely to aggregate data, postoperative morbidity stratified by technique revealed a persistent inter-technique gap but still no distinct temporal evolution in both robotic and laparoscopic arm. The annual stratified morbidity chart (Figure 5b) documented that robotic morbidity, after an initial phase of high variability between approximately 24% and 35% in 2015–2021—partly attributable to the smaller denominators of the early robotic series—stabilized toward approximately 23–25% in 2022–2025. Fully laparoscopic morbidity remained within a narrow range of approximately 18–23% throughout the entire observation period, without any directional evolution (Spearman ρ = 0.246, *p* = 0.466; CA *p* = 0.649).

Although there are no significant signs of growth or decline between the two datasets, the delta analysis (Figure 5c) confirmed that robotic morbidity exceeded laparoscopic morbidity in every year of the observation period with the sole exception of 2025, where values approached near-parity. The inter-technique gap was largest in the early and middle years of the series (approximately +5 to +15 percentage points in 2015–2021), and the smoothed delta trend showed a consistent and accelerating convergence toward zero from 2022 onward. The volume-weighted bubble chart (Figure 5d) confirmed this convergence pattern, with the robotic and laparoscopic morbidity smooth curves visually approaching each other in the 2023–2025 range, with robotic curve maintained at higher overall levels but the gap narrowing substantially relative to the 2017–2020 peak.

### 3.4. Case-Mix Evolution: Analysis on Kawaguchi-Gayet Difficulty Score over Time

To determine whether the observed inter-technique differences in clinical outcomes could be attributed, at least in part, to systematic differences in case complexity rather than to the intrinsic properties of the surgical platforms, the study included a temporal evolution of case-mix analysis based on the Kawaguchi–Gayet difficulty score.

The Kawaguchi–Gayet difficulty score distribution was analyzed for all procedures with available classification across the study period. At the aggregate level, the case mix underwent a statistically significant and near-perfectly monotonic shift toward greater complexity between 2015 and 2025. The Kruskal–Wallis test on the ordinal difficulty score across annual groups yielded H = 46.24 (*p* < 0.001), and the Spearman correlation between mean difficulty score and calendar year was ρ = 0.912 (*p* < 0.001). The Cochran–Armitage test applied separately to each difficulty level confirmed significant directional trends for all three categories: Difficulty I (*p* < 0.001), Difficulty II (*p* < 0.001), and Difficulty III (*p* < 0.001) (Table 3).

The overall distribution of Kawaguchi–Gayet difficulty categories differed significantly between robotic and fully laparoscopic approaches across the entire study period (Chi^2^, *p* = 0.018). Fully laparoscopic procedures showed a distribution of 60.2% Difficulty I, 33.1% Difficulty II, and 6.7% Difficulty III, while robotic procedures showed 58.7% Difficulty I, 32.7% Difficulty II, and 8.6% Difficulty III. The absolute inter-technique difference was 1.5 percentage points for Difficulty I, 0.4 pp for Difficulty II, and 1.9 pp for Difficulty III. Although statistically significant—a finding attributable in part to the large sample size—the magnitude of this difference was modest in absolute terms.

**Figure 6:**
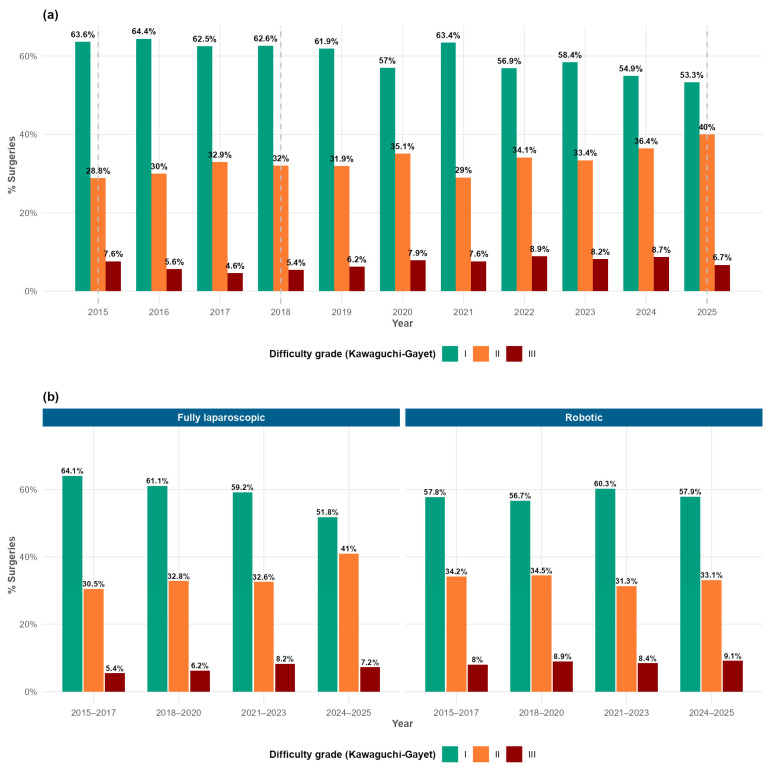
Case-mix complexity over time according to the Kawaguchi–Gayet difficulty score. (**a**) Annual distribution of difficulty grades I (low), II (intermediate), and III (high) across the full cohort (2015–2025); Cochran–Armitage trend: Grade I *p* < 0.001, Grade II *p* < 0.001, Grade III *p* < 0.001; Spearman ρ = 0.912, *p* < 0.001. (**b**) Triennial distribution of difficulty grades stratified by surgical approach (fully laparoscopic vs. robotic). For fully laparoscopic surgery, all three grades showed significant directional trends (Grade I *p* < 0.001, II *p* < 0.001, III *p* = 0.005; Spearman ρ = 0.929, *p* < 0.001). For robotic surgery, no significant trend was observed for any grade (Grade I *p* = 0.738, II *p* = 0.914, III *p* = 0.443; Spearman ρ = 0.104, *p* = 0.734). Chi-squared global comparison (laparoscopic vs. robotic, all years): *p* = 0.018. Vertical dashed lines: Morioka (2015), Southampton (2018), Brescia (2025).

The Cochran–Armitage trend tests on triennial cluster data produced diametrically opposed and statistically discriminating results for the two approaches (Figure 6). For fully laparoscopic surgery, all three difficulty levels showed statistically significant trends: Difficulty I *p* < 0.001, Difficulty II *p* < 0.001, Difficulty III *p* = 0.005. The Spearman correlation between aggregate difficulty score and calendar year for the laparoscopic series was ρ = 0.929 (*p* < 0.001). The proportion of Difficulty I laparoscopic procedures declined from 64.6% in 2015–2017 to 55.6% in 2024–2025 (−9.0 pp), while Difficulty II rose from 29.3% to 36.8% (+7.5 pp) and Difficulty III from 6.1% to 7.6% (+1.5 pp).

For robotic surgery, none of the three difficulty level trends reached statistical significance: Difficulty I *p* = 0.738, Difficulty II *p* = 0.914, Difficulty III *p* = 0.443. The Spearman correlation for the robotic series on annual data was ρ = 0.104 (*p* = 0.734). Robotic Difficulty I proportions oscillated between 57.1% and 61.5% across the four clusters without directional pattern; Difficulty II ranged from 29.1% to 36.3%; and Difficulty III from 6.6% to 9.5%. Robotic procedures exhibited a higher Difficulty III proportion than laparoscopic procedures in the earliest available cluster (8.5% vs. 6.1% in 2015–2017), and this differential persisted—with varying magnitude—across subsequent periods (6.6% vs. 5.3% in 2018–2020; 9.5% vs. 7.8% in 2021–2023; 8.8% vs. 7.6% in 2024–2025).

## 4. Discussion

The present study provides a twelve-year registry-based overview of the evolution of minimally invasive liver surgery in Italy, focusing on the progressive redistribution between fully laparoscopic and robotic liver resections and showing the potential impact of robotic surgery within the Italian context through real-world data analysis. The primary purpose of the I Go MILS registry is to collect data and track surgical activity relating to minimally invasive liver interventions within a national context, in which each participating center is included regardless of its surgical volume or surgeons’ level of expertise.

The main finding is that the I Go MILS registry captured a profound technological transition within national MILS practice: evidence highlighted that robotic liver surgery increased from approximately 10% to 48% of procedures over the study period, while pure laparoscopy proportionally declined, with the two approaches approaching a potential crossover phase in the most recent years. This observation expands the original purpose of the I Go MILS initiative, which was not only to describe the diffusion and outcomes of MILS in Italy, but also to function as a dynamic national platform able to monitor implementation, characterize practice patterns, and track temporal changes in real-world surgical activity [5,6].

The value of national registries in surgical innovation lies precisely in their ability to move beyond static outcome reporting. Similar registry- or database-based studies from France, the United States, Japan, and the Netherlands have shown that nationwide data can effectively describe the dissemination of minimally invasive liver surgery, identify temporal changes in surgical practice, and monitor safety during implementation [17,18,19,20,21,22,23,24,25,26]. In France, national analyses have documented the progressive adoption of laparoscopic and robotic liver resections over time [17,18]. In the United States, analyses from national datasets such as NSQIP and the Nationwide Inpatient Sample showed an increase in laparoscopic liver resections after the Louisville statement, with stable morbidity and mortality despite increasing use [18]. In Japan, the National Clinical Database has been used to evaluate the safe dissemination of laparoscopic liver resection in more than 27,000 cases [20]. Similarly, the Dutch Hepatobiliary Audit has provided high-quality nationwide data on the implementation of both laparoscopic and robotic liver surgery [21,22,23,24]. The present I Go MILS analysis aligns with this international registry-based framework and confirms that the role of such platforms is not limited to reporting outcomes but includes tracking how surgical practice evolves over time.

A key message of this study is that the growth of robotics should not be interpreted through a simplistic robotic-versus-laparoscopic framework. Rather, the observed trends suggest that robotic surgery has progressively entered the Italian MILS landscape as a complementary platform, preferentially used for cases of higher technical complexity. This interpretation is supported by the analysis of procedural difficulty: over the eleven-year period, the overall MILS case mix shifted toward more complex resections, with a significant decrease in low-difficulty procedures and an increase in intermediate- and high-difficulty cases. Therefore, the denominator of minimally invasive liver surgery changed substantially over time. Procedures that were previously performed through an open or hybrid approach may have progressively migrated toward minimally invasive platforms. In this context, stable morbidity and conversion rates at the population level should not be interpreted as lack of technological benefit, but rather as evidence that safety was maintained despite increasing procedural complexity.

The temporal trajectories of complexity differed between approaches. Fully laparoscopic surgery showed a progressive increase in difficulty over time, suggesting a gradual expansion of laparoscopic indications. Conversely, robotic surgery appeared to enter the registry with a more complex case mix from the early phase of adoption, without a clear further increase in difficulty over time. This pattern supports the hypothesis of a selective use of robotics for technically demanding procedures from the beginning of its national implementation. In other words, robotic liver surgery does not appear to have replaced laparoscopy across the whole MILS spectrum; instead, it seems to have occupied a specific area of indication, where the technical advantages of the platform may be most relevant.

The analysis of conversion to open surgery provides one of the most clinically meaningful findings. Although aggregate conversion rates did not show a major temporal improvement, robotic surgery consistently showed lower conversion rates than pure laparoscopy. Across the period, laparoscopic conversion ranged from 7.7% to 9.4%, whereas robotic conversion ranged from 4.2%, peaking at 7.3% and returning to 3.3% in 2025. Moreover, the annual difference between robotic and laparoscopic conversion remained in favor of robotics from 2015 onward, generally ranging from approximately −3 to −6 percentage points, although the most recent estimates should be interpreted cautiously because of smaller and incomplete denominators. Robotic surgery was associated with lower observed conversion rates; however, these unadjusted registry data do not establish an independent advantage of the robotic approach alone.

A similar nuanced interpretation is required for postoperative morbidity. Crude morbidity did not significantly decrease over time in the overall cohort, and robotic cases showed higher absolute morbidity than laparoscopic cases in several time intervals. In the following analysis, laparoscopic morbidity ranged from 17.1% to 21.7%, with an intermediate peak of 22.9% in 2022. Robotic morbidity was higher, ranging from 24% to 20%, with a peak of 27.5% in 2018. However, this should not be interpreted as evidence of inferiority of the robotic approach. The higher crude morbidity observed in the robotic group may have been influenced by differences in patient selection and procedural complexity, although this cannot be determined from the present analysis. Importantly, the most recent period showed a simultaneous reduction in robotic conversion, from 7.3% to 3.3%, and in robotic morbidity, from 25.2% to 20.2%. Although not sufficient to prove causality, this trend may reflect maturation of the robotic learning curve, refinement of indications, and increasing team experience.

This interpretation is consistent with previous reports on robotic liver surgery [27,28,29,30,31]. Studies by Ratti and colleagues have suggested that robotic and combined robotic–laparoscopic strategies may optimize intraoperative performance in major hepatectomies and complex resections [13,14]. In addition, robotic surgery has been proposed as a valuable tool for procedures requiring advanced dissection, such as lymphadenectomy for biliary tumors, where the reproducibility of open surgical principles remains essential [15]. More recently, the learning curve of robotic liver surgery has been shown to be achievable and strongly influenced by previous laparoscopic background and team-based implementation [26]. These findings support the concept that robotic adoption should be interpreted as part of the broader maturation of minimally invasive liver programs rather than as an isolated technological replacement.

The use of the Pringle maneuver further illustrates the complexity of interpreting longitudinal trends. In the overall analysis, Pringle use increased over time rather than decreasing with the adoption of robotics. When stratified by approach, both laparoscopic and robotic resections showed a significant increase in Pringle use across the considered period. In pure laparoscopy, Pringle use increased from 30.3% in 2015 to 61.8% in 2025; in robotic surgery, it increased from 16% to 51.6% over the same period. However, the robotic approach maintained lower absolute rates of Pringle use throughout almost the entire study period. This may reflect improved vascular control, stable three-dimensional visualization, instrument articulation, and enhanced precision during parenchymal transection. At the same time, the increase in Pringle use in both groups likely reflects the progressive inclusion of more complex resections within the minimally invasive domain, where preventive vascular control may be indicated independently of the surgical platform.

Overall, these findings suggest that robotic liver surgery is not replacing laparoscopy as a universal minimally invasive platform. Rather, robotics appears to be progressively defining its own area of indication within the MILS spectrum. Laparoscopy remains the high-volume platform for standard and intermediate-complexity resections, while robotics may serve as a complementary technology for anatomically difficult, technically demanding, or oncologically complex procedures [32,33,34]. This concept is clinically relevant because it shifts the interpretation of robotic adoption away from competition between platforms and toward rational allocation of the most appropriate minimally invasive approach according to case complexity, institutional expertise, and available resources. Furthermore, it is important to consider that the expanding role of artificial intelligence in conjunction with robotic systems is being more thoroughly investigated in the field of oncologic surgery. Its applications range from anatomical recognition to autonomous surgical tasks, further supporting the view that technological innovation should progressively define complementary, rather than competitive, roles within minimally invasive surgical practice [35].

The present study has several strengths. First, it is based on a national registry specifically designed to capture real-world MILS practice. Second, it evaluates temporal trends rather than providing a static comparison between approaches. Third, it integrates technological adoption, operative outcomes, and procedural complexity, allowing a more nuanced interpretation of the relationship between robotics and surgical performance. This represents one of the most important contributions of the analysis: the registry does not merely describe how many robotic resections were performed but helps explain how and why the role of robotics has changed over time.

Several limitations should be acknowledged. As with all registry-based studies, the analysis is observational and potentially affected by selection bias, center-level heterogeneity, and unmeasured confounding. The decision to perform a robotic or laparoscopic resection was not randomized and likely reflected surgeon preference, institutional resources, platform availability, case complexity, and learning-curve phase. Finally, although procedural difficulty was included in the analysis, complexity scores may not fully capture all anatomical, oncological, and technical factors influencing surgical decision-making. For these reasons, the present findings should be interpreted as a national description of evolving practice rather than as proof of causal superiority of one approach over another.

The findings reported in this study may potentially pave the way for targeted and specific follow-up research to investigate whether the robotic advantage in terms of conversion rates and the observed morbidity pattern remain consistent when stratified by type of procedure (minor vs. major hepatectomies, anatomical vs. non-anatomical resections), in order to ascertain whether the reported benefits are generalizable or depend on surgery complexity.

## 5. Conclusions

The I Go MILS registry documents a major transformation of minimally invasive liver surgery in Italy, characterized by increasing robotic adoption, proportional decline in pure laparoscopy, and progressive expansion toward more complex procedures. The stability of aggregate outcomes despite increasing complexity suggests that the national MILS community has maintained safety during a phase of technological and indication-related evolution. According to data, robotics appears to act not as a simple substitute for laparoscopy, but as a complementary platform, which may be preferentially applied to more complex resections, with suggested lower conversion rates and progressively improving outcome signals over time. These findings highlight the value of national registries in tracking trends, contextualizing innovation, and guiding the rational implementation of new technologies in hepatobiliary surgery.

## Figures and Tables

**Table 1:** Temporal overview of the I Go MILS registry (2015–2025; *N* = 8928) stratified by consensus time points. Values reported as counts (*n*), proportions (%), or absolute percentage-point differences (pp). Consensus milestones: Morioka Conference (2015); Southampton Guidelines (2018); Brescia IEGUMILS meeting (2025). These are annotated as temporal reference points and are not formally tested as intervention points. 2022 is indicated for uniformity purposes within the intervals (central year in a 7-year period) and does not represent a consensus time point. Abbreviations: CA = Cochran–Armitage trend test; n.s. = not significant (*p* ≥ 0.05); pp = percentage points.

	Consensus Time Points				CA (*p*-Value)	Spearman Rank, ρ (*p*-Value)
	Morioka	Southampton		Brescia		
	2015	2018	2022	2025	2015–2025	
***Registry Volume***						
Overall, *n* (%)	489	802	987	448	-	-
Fully Laparoscopic, *n* (%)	439 (89.8)	693 (86.4)	725 (73.5)	235 (52.5)	*p* < 0.001	−0.973 (*p* < 0.001)
Robotic, *n* (%)	50 (10.2)	109 (13.6)	262 (26.5)	213 (47.5)	*p* < 0.001	0.973 (*p* < 0.001)
***Pringle Maneuver***						
Overall, *n* (%)	141 (28.8)	358 (44.6)	605 (61.3)	277 (61.8)	*p* < 0.001	0.67 (*p* = 0.012)
Fully Laparoscopic, *n* (%)	133 (30.3)	332 (47.9)	491 (67.7)	167 (71.1)	*p* < 0.001	0.900 (*p* < 0.001)
Robotic, *n* (%)	8 (16.0)	26 (23.9)	114 (43.5)	110 (51.6)	*p* < 0.001	0.995 (*p* < 0.001)
Delta pp (Robotic–Laparoscopic)	−14.3 pp	−24 pp	−24.2 pp	−19.5 pp	-	-
***Conversion to Open Surgery***						
Overall, *n* (%)	36 (7.4)	52 (6.5)	72 (7.3)	29 (6.5)	*p* = 0.391 (n.s.)	−0.414 (*p* = 0.203, n.s.)
Fully Laparoscopic, *n* (%)	34 (7.7)	49 (7.1)	53 (7.3)	22 (9.4)	*p* = 0.882 (n.s.)	0.128 (*p* = 0.707, n.s.)
Robotic, *n* (%)	2 (4)	3 (2.8)	19 (7.3)	7 (3.3)	*p* = 0.964 (n.s.)	0.364 (*p* = 0.272, n.s.)
Delta pp (Robotic–Laparoscopic)	−3.7 pp	−4.3 pp	0 pp	−6.1 pp	-	-
***Postoperative Complications***						
Overall, *n* (%)	87 (17.8)	167 (20.8)	232 (23.5)	94 (21)	*p* = 0.478 (n.s.)	0.137 (*p* = 0.689, n.s.)
Fully Laparoscopic, *n* (%)	75 (17.1)	137 (19.8)	166 (22.9)	51 (21.7)	*p* = 0.649 (n.s.)	0.246 (*p* = 0.466, n.s.)
Robotic, *n* (%)	12 (24)	30 (27.5)	66 (25.2)	43 (20.2)	*p* = 0.045	−0.309 (*p* = 0.355, n.s.)
Delta pp (Robotic–Laparoscopic)	6.9 pp	7.7 pp	2.3 pp	−1.5 pp	-	-

**Table 2:** Perioperative and postoperative outcomes reported by international consensus conferences on minimally invasive liver surgery (Morioka 2015, Southampton 2018, Brescia 2025), stratified by surgical approach (fully laparoscopic and robotic) and summarized cumulatively for the 2015–2025 period, including operative time, hospital stay, intraoperative bleeding, postoperative complications, 30-day mortality, and Clavien–Dindo severity grading on major complications. Percentage values for the grades refer to the total number of complications recorded across the entire registry.

	Consensus Time Points				
	Morioka	Southampton		Brescia	
	2015	2018	2022	2025	2015–2025
*Perioperative Outcomes*					
*Total, n*	489	802	987	448	8928
*Fully laparoscopic, n*	439	693	752	235	7092
*Robotic, n*	50	109	262	213	1836
***Patients with previous liver surgery, n (%)***					
*Total, n (%)*	101 (20.7)	222 (27.7)	283 (28.7)	137 (30.6)	2452 (27.5)
*Fully laparoscopic, n (%)*	93 (21.2)	194 (28.0)	215 (29.7)	76 (32.3)	1960 (27.6)
*Robotic, n (%)*	8 (16.0)	28 (25.7)	68 (26.0)	61 (28.6)	492 (26.8)
***Mean Operative time (hours)***					
*Total*	3.61	4.2	4.84	4.79	4.53
*Fully laparoscopic*	3.48	4.15	4.82	4.82	4.45
*Robotic*	4.72	4.53	4.88	4.76	4.85
***Mean Hospital stay (days)***					
*Total*	5.78	6.09	6.19	5.97	6.30
*Fully laparoscopic*	5.80	5.98	6.20	5.90	6.28
*Robotic*	5.58	6.79	6.16	6.04	6.37
***Surgeries with intraoperative bleeding, n (%)***					
*Total*	435 (89.0)	738 (92.0)	893 (90.5)	380 (84.8)	8085 (90.6)
*Fully laparoscopic*	388 (88.4)	632 (91.2)	640 (88.3)	206 (87.7)	6366 (89.8)
*Robotic*	47 (94.0)	106 (97.2)	231 (96.6)	174 (81.7)	1719 (93.6)
***Mean volume of bleeding (mL)***					
*Total*	194	178	222	186	211
*Fully laparoscopic*	195	171	219	210	206
*Robotic*	185	226	231	160	229
***Postoperative Outcomes***					
***Biliary Fistula, n (%)***					
*Total*	9 (1.8)	13 (1.6)	24 (2.4)	12 (2.7)	238 (2.7)
*Fully laparoscopic*	8 (1.8)	11 (1.6)	15 (2.1)	6 (2.6)	167 (2.4)
*Robotic*	1 (2.0)	2 (1.8)	9 (3.4)	6 (2.8)	71 (3.9)
***Wound Infections, n (%)***					
*Total*	7 (1.4)	7 (0.9)	10 (1.0)	0	75 (0.8)
*Fully laparoscopic*	7 (1.6)	6 (0.9)	6 (0.8)	0	55 (0.8)
*Robotic*	0	1 (0.9)	4 (1.5)	0	20 (1.1)
***Hemorrhagic events, n (%)***					
*Total*	7 (1.4)	2 (0.2)	22 (2.2)	9 (2.0)	138 (1.5)
*Fully laparoscopic*	7 (1.6)	2 (0.3)	13 (1.8)	3 (1.3)	107 (1.5)
*Robotic*	0	0	9 (3.4)	6 (2.8)	31 (1.7)
***30-day mortality rate, %***					
*Total*	0	0.4	0.3	0.4	0.3
*Fully laparoscopic*	0	0.3	0.3	0.4	0.3
*Robotic*	0	0.9	0.4	0.5	0.7
***Clavien–Dindo grades for major complications***					
***Grade I, n (%)***					
*Total*	87 (53.0)	167 (49.4)	232 (48.2)	93 (53.1)	1936 (49.1)
*Fully laparoscopic*	75 (52.8)	137 (48.8)	166 (49.6)	50 (54.3)	1458 (49.7)
*Robotic*	12 (54.5)	30 (52.6)	66 (45.2)	43 (51.8)	478 (47.6)
***Grade II, n (%)***					
*Total*	57 (34.8)	123 (36.4)	186 (38.7)	60 (34.3)	1443 (36.6)
*Fully laparoscopic*	48 (33.8)	102 (36.3)	132 (39.4)	33 (35.9)	1069 (36.4)
*Robotic*	9 (40.9)	21 (36.8)	54 (37.0)	27 (32.5)	374 (37.3)
***Grade III, n (%)***					
*Total*	16 (9.8)	41 (12.1)	52 (10.8)	17 (9.7)	452 (11.5)
*Fully laparoscopic*	15 (10.6)	36 (12.8)	32 (9.6)	8 (8.7)	332 (11.3)
*Robotic*	1 (4.5)	5 (8.8)	20 (13.7)	9 (10.8)	120 (12.0)
***Grade IV, n (%)***					
*Total*	4 (2.4)	4 (1.2)	9 (1.9)	2 (1.1)	81 (2.1)
*Fully laparoscopic*	4 (2.8)	4 (1.4)	3 (0.9)	0	60 (2.0)
*Robotic*	0	0	6 (4.1)	2 (2.4)	21 (2.1)
***Grade V, n (%)***					
*Total*	0	3 (0.9)	2 (0.4)	3 (1.7)	27 (0.7)
*Fully laparoscopic*	0	2 (0.7)	2 (0.6)	1 (1.1)	16 (0.5)
*Robotic*	0	1 (1.8)	0	2 (2.4)	11 (1.1)

**Table 3:** Overall evolution of Kawaguchi–Gayet difficulty grade (I, II and III) stratified by consensus time points. Values reported as counts (*n*), proportions (%). Consensus milestones: Morioka Conference (2015); Southampton Guidelines (2018); Brescia IEGUMILS meeting (2025). These are annotated as temporal reference points and are not formally tested as intervention points. 2022 is indicated for uniformity purposes within the intervals (central year in a 7-year period) and does not represent a consensus time point. Abbreviations: CA = Cochran–Armitage trend test.

	Consensus Time Points				CA Test ( *p*-Value)
	Morioka	Southampton		Brescia	
	2015	2018	2022	2025	2015–2025
Grade I—Low difficulty, *n* (%)	311 (63.6)	502 (62.6)	562 (56.9)	239 (53.3)	*p* < 0.001
Grade II—Intermediate difficulty, *n* (%)	141 (28.8)	257 (32)	337 (34.1)	179 (40)	*p* < 0.001
Grade III—High difficulty, *n* (%)	37 (7.6)	43 (5.4)	88 (8.9)	30 (6.7)	*p* < 0.001

## Data Availability

The data presented in this study are available upon reasonable request from the corresponding author due to privacy reasons.

## Abbreviations

MILS	Minimally Invasive Liver Surgery
CA	Cochran–Armitage Trend Test
n.s.	Not Significant
pp	Percentage Points
LOESS	Locally Estimated Scatterplot Smoothing
CI	Confidence Interval
NSQIP	National Surgical Quality Improvement Program

## References

[1] Wakabayashi G., Cherqui D., Geller D.A., Buell J.F., Kaneko H., Han H.S., Asbun H., O’Rourke N., Tanabe M., Koffron A.J. (2015). Recommendations for laparoscopic liver resection: A report from the second international consensus conference held in Morioka. Ann. Surg..

[2] Abu Hilal M., Aldrighetti L., Dagher I., Edwin B., Troisi R.I., Alikhanov R., Aroori S., Belli G., Besselink M., Briceno J. (2018). The Southampton Consensus Guidelines for laparoscopic liver surgery: From indication to implementation. Ann. Surg..

[3] Abu Hilal M., Hoogteijling T.J., Edwin B., Dagher I., D’Hondt M., Marques H.P., Swijnenburg R.J., Boggi U., Sucandy I., Ferrero A. (2025). The Brescia internationally validated European guidelines on minimally invasive liver surgery. Br. J. Surg..

[4] Liu R., Abu Hilal M., Wakabayashi G., Han H.S., Palanivelu C., Boggi U., Hackert T., Kim H.J., Wang X.Y., Hu M.G. (2023). International experts consensus guidelines on robotic liver resection in 2023. World J. Gastroenterol..

[5] Aldrighetti L., Ratti F., Cillo U., Ferrero A., Ettorre G.M., Guglielmi A., Giuliante F., Calise F., Italian Group of Minimally Invasive Liver Surgery (2017). Diffusion, outcomes and implementation of minimally invasive liver surgery: A snapshot from the I Go MILS Registry. Updat. Surg..

[6] Ratti F., Ferrero A., Guglielmi A., Cillo U., Giuliante F., Mazzaferro V., De Carlis L., Ettorre G.M., Gruttadauria S., Di Benedetto F. (2023). Ten years of Italian mini-invasiveness: The I Go MILS registry as a tool of dissemination, characterization and networking. Updat. Surg..

[7] Kamel M.K., Tuma F., Keane C.A., Blebea J. (2022). National Trends and Perioperative Outcomes of Robotic-assisted Hepatectomy in the USA: A Propensity-score Matched Analysis from the National Cancer Database. World J. Surg..

[8] Ciria R., Berardi G., Alconchel F., Briceño J., Choi G.H., Wu Y.M., Sugioka A., Troisi R.I., Salloum C., Soubrane O. (2022). The impact of robotics in liver surgery: A worldwide systematic review and short-term outcomes meta-analysis on 2,728 cases. J. Hepato-Biliary-Pancreat. Sci..

[9] Liu Q., Zhang W., Zhao J.J., Syn N.L., Cipriani F., Alzoubi M., Aghayan D.L., Siow T.F., Lim C., Scatton O. (2023). International robotic and laparoscopic liver resection study group investigators. Propensity-score Matched and Coarsened-exact Matched Analysis Comparing Robotic and Laparoscopic Major Hepatectomies: An International Multicenter Study of 4822 Cases. Ann. Surg..

[10] Beard R.E., Tsung A. (2017). Minimally Invasive Approaches for Surgical Management of Primary Liver Cancers. Cancer Control.

[11] Wang P., Zhang D., Huang B., Zhou W.H., Wang C.S., Zhao S.Y., Su S., Jiang X.Z. (2025). Robotic versus laparoscopic hepatectomy: Meta-analysis of propensity-score matched studies. BJS Open.

[12] Kitisin K., Packiam V., Bartlett D.L., Tsung A. (2011). A current update on the evolution of robotic liver surgery. Minerva Chir..

[13] Zwart M.J.W., Görgec B., Arabiyat A., Nota C.L.M., van der Poel M.J., Fichtinger R.S., Berrevoet F., van Dam R.M., Aldrighetti L., Fuks D. (2022). Pan-European survey on the implementation of robotic and laparoscopic minimally invasive liver surgery. HPB.

[14] Aldrighetti L., Catena M., Ratti F., Cipriani F. (2022). Maximizing performance in complex minimally invasive surgery of the liver: The RoboLap approach. J. Gastrointest. Surg..

[15] Ratti F., Marino R., Ingallinella S., Clocchiatti L., Corallino D., Catena M., Aldrighetti L. (2023). Robo-Lap approach optimizes intraoperative outcomes in robotic left and right hepatectomy. J. Soc. Laparosc. Robot. Surg..

[16] Ratti F., Cipriani F., Ingallinella S., Tudisco A., Catena M., Aldrighetti L. (2023). Robotic approach for lymphadenectomy in biliary tumors: The missing ring between the benefits of laparoscopic and reproducibility of open approach. Ann. Surg..

[17] Kawaguchi Y., Fuks D., Kokudo N., Gayet B. (2018). Difficulty of Laparoscopic Liver Resection: Proposal for a New Classification. Ann. Surg..

[18] Farges O., Goutte N., Dokmak S., Bendersky N., Falissard B., ACHBT French Hepatectomy Study Group (2014). How surgical technology translates into practice: The model of laparoscopic liver resections performed in France. Ann. Surg..

[19] Deyrat J., Fuks D., Murris J., Lanoy E., Nassar A., Dhote A., Marchese U., Mallet V., Katsahian S., Gaillard M. (2024). Evolution of minimally invasive liver surgery in France over the last decade: A nationwide analysis. Surg. Endosc..

[20] He J., Amini N., Spolverato G., Hirose K., Makary M., Wolfgang C.L., Weiss M.J., Pawlik T.M. (2015). National trends with a laparoscopic liver resection: Results from a population-based analysis. HPB.

[21] Ban D., Tanabe M., Kumamaru H., Nitta H., Otsuka Y., Miyata H., Kakeji Y., Kitagawa Y., Kaneko H., Wakabayashi G. (2021). Safe dissemination of laparoscopic liver resection in 27,146 cases between 2011 and 2017 from the National Clinical Database of Japan. Ann. Surg..

[22] van der Poel M.J., Fichtinger R.S., Bemelmans M., Bosscha K., Braat A.E., de Boer M.T., Dejong C.H.C., Doornebosch P.G., Draaisma W.A., Gerhards M.F. (2019). Implementation and outcome of minor and major minimally invasive liver surgery in the Netherlands. HPB.

[23] Görgec B., Zwart M., Nota C.L., Bijlstra O.D., Bosscha K., de Boer M.T., de Wilde R.F., Draaisma W.A., Gerhards M.F., Liem M.S. (2023). Implementation and outcome of robotic liver surgery in the Netherlands: A nationwide analysis. Ann. Surg..

[24] Pilz da Cunha G., Sijberden J.P., van Dieren S., Gobardhan P., Lips D.J., Terkivatan T., Marsman H.A., Patijn G.A., Leclercq W.K.G., Bosscha K. (2024). Robotic versus laparoscopic liver resection: A nationwide analysis from the Dutch Hepato Biliary Audit. Ann. Surg..

[25] de Graaff M.R., Klaase J.M., Dulk M.D., Buis C.I., Derksen W.J.M., Hagendoorn J., Leclercq W.K.G., Liem M.S.L., Hartgrink H.H., Swijnenburg R.J. (2024). Outcomes of liver surgery: A decade of mandatory auditing in the Netherlands. Eur. J. Surg. Oncol..

[26] Ratti F., Ingallinella S., Catena M., Corallino D., Marino R., Aldrighetti L. (2025). Learning curve in robotic liver surgery: Easily achievable, evolving from laparoscopic background and team-based. HPB.

[27] Guerra F., Guadagni S., Pesi B., Furbetta N., Di Franco G., Palmeri M., Annecchiarico M., Eugeni E., Coratti A., Patriti A. (2019). Outcomes of robotic liver resections for colorectal liver metastases. A multi-institutional analysis of minimally invasive ultrasound-guided robotic surgery. Surg. Oncol..

[28] Rocca A., Scacchi A., Cappuccio M., Avella P., Bugiantella W., De Rosa M., Costa G., Polistena A., Codacci-Pisanelli M., Amato B. (2021). Robotic surgery for colorectal liver metastases resection: A systematic review. Int. J. Med. Robot..

[29] Safiejko K., Pedziwiatr M., Pruc M., Tarkowski R., Juchimiuk M., Domurat M., Smereka J., Anvarov K., Sielicki P., Kurek K. (2024). Robotic versus Laparoscopic Liver Resections for Colorectal Metastases: A Systematic Review and Meta-Analysis. Cancers.

[30] Mkabaah L.B., Davey M.G., Kerin E.P., Ryan O.K., Ryan E.J., Donnelly M., Ahmed O., McEntee G.P., Conneely J.B., Donlon N.E. (2025). Comparing Open; Laparoscopic and Robotic Liver Resection for Metastatic Colorectal Cancer-A Systematic Review and Network Meta-Analysis. J. Surg. Oncol..

[31] Fagenson A.M., Gleeson E.M., Pitt H.A., Lau K.N. (2021). Minimally Invasive Hepatectomy in North America: Laparoscopic Versus Robotic. J. Gastrointest. Surg..

[32] Abdelhadi S., El-Ahmar M., Abbasi Dezfouli S., Vedder K., Hermann M., Orth V., Halawa M., Moennichs M., Reissfelder C., Sandra-Petrescu F. (2025). Robotic-assisted versus laparoscopic versus open liver resection: Comparison of postoperative outcomes according to the IWATE difficulty score. Surg. Endosc..

[33] von Kroge P.H., Fard-Aghaie M., Afshar-Bakshloo K., Izbicki J.R., Hackert T., Li J., Schmelzer I., Nickel F., Ghadban T., Heumann A. (2026). Comparison of laparoscopic and robotic liver surgery: A German real-world analysis. Surg. Endosc..

[34] Schmelzle M., Feldbrügge L., Ortiz Galindo S.A., Moosburner S., Kästner A., Krenzien F., Benzing C., Biebl M., Öllinger R., Malinka T. (2022). Robotic vs. laparoscopic liver surgery: A single-center analysis of 600 consecutive patients in 6 years. Surg. Endosc..

[35] Malerba S., Vladimirov M., Goyal A., Dulskas A., Baušys A., Cwalinski T., Girnyi S., Skokowski J., Duka R., Molchanov R. (2026). Artificial Intelligence Applications in Gastric Cancer Surgery: Bridging Early Diagnosis and Responsible Precision Medicine. J. Clin. Med..

